# Walking ability during daily life in patients with osteoarthritis of the knee or the hip and lumbar spinal stenosis: a cross sectional study

**DOI:** 10.1186/1471-2474-11-233

**Published:** 2010-10-12

**Authors:** Corinna C Winter, Mirko Brandes, Carsten Müller, Tim Schubert, Michael Ringling, Axel Hillmann, Dieter Rosenbaum, Tobias L Schulte

**Affiliations:** 1 Department of Orthopedics and Tumor Orthopedics, University Hospital Muenster, Albert-Schweitzer-Str. 33, 48149 Münster, Germany; 2 Institute of Sports Science, University of Bremen, Badgasteiner-Str. 1, 28359 Bremen, Germany; 3 Orthopedic Department, Klinikum Ingolstadt, Krumenauerstr. 25, 85021 Ingolstadt, Germany

## Abstract

**Background:**

Degenerative musculoskeletal disorders are among the most frequent diseases occurring in adulthood, often impairing patients' functional mobility and physical activity. The aim of the present study was to investigate and compare the impact of three frequent degenerative musculoskeletal disorders -- knee osteoarthritis (knee OA), hip osteoarthritis (hip OA) and lumbar spinal stenosis (LSS) -- on patients' walking ability.

**Methods:**

The study included 120 participants, with 30 in each patient group and 30 healthy control individuals. A uniaxial accelerometer, the StepWatch™ Activity Monitor (Orthocare Innovations, Seattle, Washington, USA), was used to determine the volume (number of gait cycles per day) and intensity (gait cycles per minute) of walking ability. Non-parametric testing was used for all statistical analyses.

**Results:**

Both the volume and the intensity of walking ability were significantly lower among the patients in comparison with the healthy control individuals (p < 0.001). Patients with LSS spent 0.4 (IQR 2.8) min/day doing moderately intense walking (>50 gait cycles/min), which was significantly lower in comparison with patients with knee and hip OA at 2.5 (IQR 4.4) and 3.4 (IQR 16.1) min/day, respectively (p < 0.001). No correlations between demographic or anthropometric data and walking ability were found. No technical problems or measuring errors occurred with any of the measurements.

**Conclusions:**

Patients with degenerative musculoskeletal disorders suffer limitations in their walking ability. Objective assessment of walking ability appeared to be an easy and feasible tool for measuring such limitations as it provides baseline data and objective information that are more precise than the patients' own subjective estimates. In everyday practice, objective activity assessment can provide feedback for clinicians regarding patients' performance during everyday life and the extent to which this confirms the results of clinical investigations. The method can also be used as a way of encouraging patients to develop a more active lifestyle.

## Background

Degenerative musculoskeletal disorders are among the most common diseases in adulthood and account for a high percentage of cases of in-patient treatments, temporary disability, inability to work and early retirement [1,2]. The prevalence of degenerative diseases increases with age, and current demographic changes are likely to exacerbate the associated problems for modern societies. At the same time, elderly people nowadays have high standards in relation to their mobility, independence, quality of life and ability to take part in social life. The ability to walk is considered to be essential for most activities of daily living [3].

In the field of orthopedics the most common and most frequent conditions leading to major restrictions of walking ability in patients over the age of 55 years are osteoarthritis of the hip and knee and spinal claudication due to lumbar spinal stenosis associated with osteoarthritis of the zygapophyseal joints [4]. A major proportion of orthopedic surgeries are carried out in these patients in order to restore mobility [5]. Typical signs of OA are pain, joint stiffness and reduced walking ability. The knee and the hip joints differ substantially in their anatomical structure, ligamentous stabilization and biomechanical characteristics. In addition, different factors may lead to the development of OA in the two joints. It is therefore relevant to investigate whether the site of OA has an influence on patients' walking ability. Lumbar spinal stenosis (LSS) has an annual incidence of about five per 100,000 population and is the most common reason for spinal surgery [6]. From the pathophysiological point of view, LSS is similar to OA in the peripheral joints as it results from bony hypertrophy of osteoarthritic zygapophyseal joints, degenerative changes and hypertrophy of the ligamenta flava and degeneration of the intervertebral disc. In patients with LSS, pain is caused by the compression of the nerve root leading to neurogenic claudication as a cardinal symptom of the condition. The pain radiates particularly into the back and the legs and becomes more severe as the walking distance increases. These symptoms are not typically seen in patients with knee or hip OA. Despite the different pathological mechanisms underlying the conditions, limited mobility is the major symptom in all three groups of patients.

The aim of the present study was to use an objective method to quantify and compare the impact of knee OA, hip OA and LSS on patients' walking ability. Objective measurements of the extent of disease-induced restrictions in patients' everyday life were carried out and can be used as a basis for future studies, e.g., intervention studies.

Subjective assessments of patients' physical activity using questionnaires or self-reporting have been found to be inconsistent. Accelerometry, using devices worn on the body that are able to detect the acceleration of the body or parts of it in order to assess activity patterns, has been shown to provide more accurate activity measurements [7]. In contrast to laboratory situations, in which only a snapshot of the patient's walking ability can be obtained [8] accelerometry provides information about activities during daily life. Only objective data during daily life were assessed in the present study. The same device was used for all measurements, allowing direct comparisons of the impact of various diseases on walking ability.

The main research questions addressed in the present study were:

• What is the extent of daily walking ability in normal everyday conditions for patients with the most common degenerative orthopedic diseases?

• How do these data compare with a healthy cohort of similar age and gender distribution?

• Are there any differences between patients suffering from degenerative disease of the spine, the hip or the knee? Are there particularly severe limitations affecting one of these groups?

• Does the measurement technique used represent a practical tool for assessing patients with degenerative musculoskeletal diseases?

## Methods

### Patients

The study included 120 participants, 30 patients each with knee OA, hip OA and LSS and 30 healthy control individuals with a comparable age and gender distribution (Table 1). All of the patients had radiographic and clinical signs that were pathognomonic for their diseases. In patients with OA the grade of OA was 3 or higher according to the Kellgren-Lawrence system. Patients with LSS also had high disease grades. All of the patients were suffering from severe symptoms for which conservative treatment had failed, and they were all scheduled for either primary endoprosthetic joint replacement or decompressive spinal surgery. The decision to carry out surgery had been made by experienced orthopedic surgeons, totally independent from this investigation. The control individuals had no history of musculoskeletal disorders and reported no knee, hip, or back pain and no walking restrictions. Exclusion criteria for all participants consisted of peripheral arterial diseases, rheumatic diseases, systemic muscular diseases, fractures in the lower extremities and cardiac, pulmonary and circulatory diseases severely affecting the patients' mobility. Informed written consent to participate in the investigation was obtained from all of the participants. The study was approved by the Ethics Committee at Muenster University and was conducted in accordance with the Helsinki Declaration of 1975, revised in 1983.

**Table 1:** Anthropometric and demographic data for the patients and control group

	Knee OA (n = 30) MD, IQR	Hip OA (n = 30) MD, IQR	LSS (n = 30) MD, IQR	CON (n = 30) MD, IQR	p-values
Age (y)	63.2 , 5.1	61.0, 20.0	66.3, 7.7	63.5, 9.8	0.35

Height (m)	1.67, 0.10	1.69, 0.15	1.73, 0.12	1.70, 0.13	0.16

Weight (kg)	82.0, 19.5	78.0, 21.0	90.0, 17.0	75.0, 14.8	0.08

BMI (kg/m^2^)	29.8, 5.9	26.0, 3.8	28.1, 4.5	25.9, 3.6	0.27

Overweight (BMI > 25; n)	12	10	15	13	

Obese (BMI > 30; n)	13	2	10	4	

**female/male**	15/15	16/14	12/18	15/15	

LSS = Lumbar Spinal Stenosis, CON = Control group, MD = Median, IQR = Inter Quartile Range

A total of 128 individuals were invited to participate in the study. Seven patients did not agree to participate, and the data for one patient could not be evaluated as the measurement period was too short. Reasons given for non-participation were either that the patients had no interest in the study or that they expected unfavorable results as their walking was severely restricted. Four patients with LSS, two patients with knee OA and one patient with hip OA did not agree to participate.

### Assessment of Walking Ability

Walking ability before surgery was investigated on seven consecutive days. The number of gait cycles (one gait cycle consists of two steps) was determined with the StepWatch 3™ Activity Monitor (SAM; Orthocare Innovations, Seattle, WA, USA). The device is a small (7.5 × 5 × 2 cm), light-weight (43 g), uniaxial accelerometer. It is attached to the ankle with an elastic strap and is unobtrusive for the user. An internal clock records information about timing and bouts of activities.

The monitor has been validated in several studies and has been reported to have a 99% accuracy rate in detecting gait cycles per time interval [9,10]. Before being attached to start data collection, the unit was programmed to adjust for the patient's individual height, walking speed (slow, normal, fast) and range of walking speeds. It is not possible to manipulate the device, e.g., by shaking or moving without any actual walking activity. The SAM monitors and saves data over several weeks and operates without providing the user with any feedback, thereby minimizing test bias. It was programmed to store data at intervals of one minute; therefore, data was available for every minute of the observation period. The volume and the intensity of activity were assessed. Volume was defined as the total number of gait cycles per day or per hour. To take the potential influence of different wearing times into account, gait cycles were also evaluated per hour of the time the device was actually worn. Intensity of activity was defined by the number of gait cycles per minute. Moderately-intense aerobic physical activity is part of accepted recommendations for a healthy lifestyle [11]. Translated into walking, this level was achieved by performing at least 100 steps per minute [12] or 50 gait cycles per minute. Continuous brisk walking was required in order to achieve this level of activity [11]. The results are given as both the percentage of overall activity spent at the moderate level and as the number of minutes spent at this level.

The device was worn during waking hours with the wearing periods also being documented in a daily log list. Measurements were carried out 35.3 ± 5.2 days before surgery. The participants wore the SAM for a mean of 6.5 ± 0.8 days (min. 5 days, max. 10 days). The average daily wearing time was 13.8 ± 1.5 hours (min. 10.1 hours, max. 17.1 hours).

During the week SAM monitoring was being carried out pain was additionally assessed by having the patients rate it on the following scale as used in the Short Form 36 questionnaire: no pain, very little pain, little pain, moderate pain, severe pain, unbearable pain.

### Data Analysis

On the assumption that 2000 gait cycles per day would represent a clinically relevant difference with a standard deviation of 2000 gait cycles per day, power calculation showed that 27 patients would have to be recruited for a substantial effect (d = 1) to be observed with α = 0.008 (after Bonferroni correction) and β = 0.80. Allowing for possible drop-outs, it was decided to include 120 individuals with 30 in each group.

Descriptive statistics were calculated to compare the demographic and anthropometric data, as well as the activity behavior of the sample populations. The Kolmogorov-Smirnov test was used to test for normal distribution. Data did not fulfill requirements for parametric testing; therefore, differences between groups were assessed with the Kruskal-Wallis H-test and the Mann-Whitney U-test. All results are given as medians and interquartile ranges (IQR). The level of statistical significance was set at p < 0.008 after Bonferroni correction for multiple post-hoc pair-wise comparisons. Spearman correlation coefficients were determined. The Statistical Package for the Social Sciences (SPSS, version 15.0) was used for all statistical analyses.

## Results

**Figure 1:**
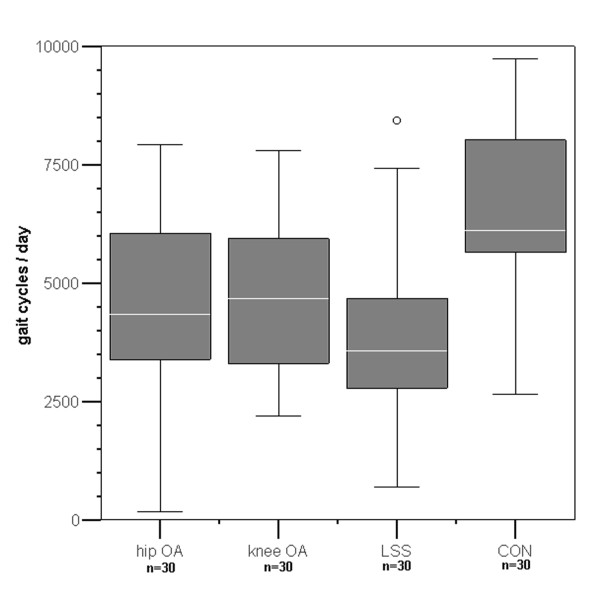
**Gait cycles per day of patients and control group **OAK = Knee osteoarthritis, OAH = Hip osteoarthritis, LSS = Lumbar Spinal Stenosis, CON = Control group, o outlier

Hourly gait cycles were highly correlated with the gait cycles per day (r = 0.95, p < 0.01). Therefore, the analysis of gait cycles per hour did not provide any further information and the volume of activity will be reported as gait cycles per day only. The volume of activity (assessed as gait cycles per day) was significantly lower in all of the patients in comparison with the healthy control group (p < 0.001; Table 2, Figure 1). Patients with knee OA reached 76.4% of the control groups' median values, patients with hip OA 65.3% and patients with LSS 58.2%. There were no statistically significant differences between the patient groups.

**Table 2:** Data for walking ability in patients and controls

	Knee OA (n = 30) MD, IQR	p-value vs CON	Hip OA (n = 30) MD, IQR	p-value vs. CON	LSS (n = 30) MD, IQR	p-value vs CON	CON (n = 30) MD, IQR	p-value knee-hip	p-value knee-LSS	p-value hip-LSS
GC/day	4675, 2575	0.001	3994, 2640	0.001	3564, 1839	0.001	6119, 2234	0.67	0.03	0.112

GC/hour	405, 206	0.007	317, 211	0.004	271, 150	0.001	450, 157	0.575	0.02	0.127

High Int. (%)	0.8, 1.6	0.001	1.0, 4.9	0.001	0.1, 1.0	0.001	3.5, 4.1	0.450	0.007	0.005

**Min > 50**	2.5, 4.4	0.001	3.4, 16.1	0.006	0.4, 2.8	0.001	15.8, 15.6	0.670	0.005	0.003

GC = Gait cycles, Int = Intensity, LSS = Lumbar Spinal Stenosis, CON = Control group, MD = Median, IQR = Inter Quartile Range,

Min > 50 = minutes per day spend above 50 gait cycles per minute

The number of minutes spent above 50 gait cycles per minute and the percentage spent at this level of activity were significantly lower in all three patient groups compared to healthy subjects (p < 0.001; Table 2). Patients with LSS spent significantly less time performing moderate physical activity and the percentage of activity spent at this level was significantly less compared to both patient groups with OA (Table 2). No significant differences were detected between patients with hip or knee OA.

None of the patients reported any problems in using the device or restrictions resulting from the measurement. None of the patients canceled the measurement, one patient applied the device too late and measurement duration was too short. Data for all the other patients were evaluable and were used for analysis.

There were no significant differences between the groups with regard to pain which did not correlate with the activity data. Furthermore, neither high nor significant correlations between step activity data and anthropometric data, i.e. age, height, weight and body mass index (BMI) could be detected (Table 3).

**Table 3:** Spearman Correlation Coefficients for anthropometric data and activity data (all patients)

	age	height	weight	BMI
GC/day	0.158 (n.s.)	0.205 (n.s.)	0.102 (n.s.)	-0.069 (n.s.)

GC/hour	0.145 (n.s.)	0.102 (n.s.)	0.112 (n.s.)	-0.045 (n.s.)

High INT	0.017(n.s.)	-0.82 (n.s.)	-0.43 (n.s.)	0.068 (n.s.)

Min > 50	0.087(n.s.)	-0.73 (n.s.)	-0.028 (n.s.)	0.039 (n.s.)

GC = Gait cycles, INT = Intensity, LSS = Lumbar Spinal Stenosis, CON = Control group, SD = Standard deviation, Min > 50 = minutes spend above 50 gait cycles per minute

## Discussion

Patients with degenerative orthopaedic diseases were found to have considerable reductions in the volume and the intensity of walking ability in comparison to healthy control individuals of comparable age. With regard to the volume and intensity of activity patients with knee and hip OA were comparable with one another. While patients with knee OA had a slightly greater volume of activity, patients with hip OA were found to spend slightly more time on moderately intense walking. However, the differences were minor and not significant and did not appear to be clinically relevant. Possible influences of anatomical and biomechanical differences between the knee and the hip joints and of different disease mechanisms were not evident in the patients studied and were not subject of the study.

Patients with LSS were found to have the greatest restrictions in their walking ability, particularly in relation to moderately intense walking. These differences suggest that disease-specific symptoms of LSS, resulting from nerve compression and leading to spinal claudication, limit patients' ability to walk to a greater extent than the symptoms of knee or hip OA do. All of the patients were scheduled to undergo appropriate surgeries as conservative treatment strategies had not led to sufficient improvement in their symptoms. The patients' subjective perception of the risk associated with the surgical interventions might have contributed to some of the observed differences. Endoprosthetic joint replacement is nowadays a generally-accepted surgical procedure that is not especially feared by patients. However, it is possible that spinal surgery may be regarded with more skepticism due to fear of adverse consequences. Patients with LSS may therefore wait longer and present with more severe restrictions before agreeing to undergo surgery - although the intensity of pain did not differ between groups and did not correlate with activity data. Assessment is particularly valuable in patients with LSS, to allow objective quantification of the actual extent of restrictions and to address this topic with the patient. These patients may need special attention in order to encourage walking and physical activity before and after surgery.

Generally recognized risk factors for degenerative musculoskeletal diseases, i.e., advanced age and a high BMI were also present in the patients included in the present study [2,13-16]. However, there were no significant differences either with regard to age or BMI and no correlations between these factors and the patients' activity data were observed.

The device used in the present study appeared to be an easily applicable tool for measuring walking ability in patients with degenerative musculoskeletal disorders. Adherence to measurement process was very good as the wearing times recorded by the SAM were confirmed by the times noted in the log-list. In addition, the minimum wear time was five days per week. Only seven patients (four with LSS) did not agree to participate. All participating patients accepted the device and no application errors or malfunctions were observed. The results therefore were unlikely to have been affected by any adherence problems.

The technique used here can be recommended for further research on mobility in patients suffering from orthopedic problems. Impaired mobility is also a common symptom in non-orthopedic diseases, e.g. vascular claudication and neurological disorders and the technique could also be used in those fields. Practical applications might include monitoring the rehabilitation process of patients by carrying out repeated measurements and comparing different time points, e.g., before and after intervention. The technique could also be used to compare different intervention strategies or as an intervention in itself by encouraging the patients to reach certain levels of activity. However, further research will be required in order to assess the method's performance characteristics before it is used in longitudinal studies.

Co-morbid conditions are quite common in the age group of the patients investigated in the study and reflect the typical situation in many older patients. Most co-morbidities were excluded in this study in order to focus on the actual impact of OA or LSS. However, possible co-morbidities that were not explicitly excluded might still have influenced the results. A different approach might have involved co-morbidities as typical confounding factors and adjusting the data to take their effects into account. This would have provided more detailed and more realistic information about these patients but would have resulted in very small subgroups, creating statistical problems.

In daily clinical practice objective activity measurements provide valuable additional information about what patients are able to perform during everyday life and in their own familiar surroundings. Performance tests such as timed walking tests in patients with OA or treadmill tests in patients with LSS, are well-established and are frequently used in clinical practice. These tests are conducted in laboratory settings and provide only a snap-shots of the patient [8] to assess what the patient is capable of; they provide no information about what a patient actually does at home in real-life conditions.

Only one previous study has compared patients with knee OA, hip OA and LSS: Three groups were evaluated with self-reported quality of life and but did not reveal any significant differences before surgery [17]. Activity restrictions measured by accelerometry have been reported in patients with early OA of the knee [18] and in patients with low back pain [16]. These findings were confirmed by the present study. However, direct comparisons of results of different studies are difficult due to different measurement devices and different stages of the diseases.

Most previous studies on activity in patients with OA or LSS used self-reports to determine the level of activity. This approach is influenced by each individual's own perceptions and interpretations and can provide valuable information when rating a patient's state of well-being. However, self-reports are considered as an insufficient and imprecise approach for quantitative activity assessments [19]. Most questionnaires do not assess low intensity levels which are quite typical in patient populations [19]. In previous studies accelerometry has been shown to be a good and valid method here [7]. Comparisons between a patient's self-reported activity and actual activity and correlations between actual activity and self-reported well-being could be valuable. This would provide information about whether or not a patient's perception is adequate. However, such comparisons were not the subject of the present study.

To the best of our knowledge, this is the first study that has used a single measurement device to compare the impact of different degenerative musculoskeletal diseases with respect to walking ability. The study therefore focused on providing descriptive information and baseline data for normal conditions in everyday life.

Limitations of the present study were the rather inhomogeneous sample of patients in the different groups and between the different groups, with respect to disease progression, age and anthropometric data. Furthermore, the exclusion of co-morbidities might have led to a biased patient sample and allowed no conclusions about the impact of confounders. Furthermore, no self-reported data was assessed for a comparison with objective data.

## Conclusions

The present study provides basic activity data for three distinct groups of patients with degenerative musculoskeletal diseases. Considerably limited walking ability was revealed in patients with knee OA, hip OA and LSS. Patients with LSS experienced the greatest restrictions, probably due to more severe symptoms. No relevant correlations between the anthropometric or demographic data and the activity data were observed.

The device used allowed objective assessment of walking ability and appeared to be an easy and feasible tool for the determination of limitations. In everyday practice, objective activity assessment can provide feedback for clinicians regarding patients' performance during everyday life and the extent to which this confirms results of clinical investigations. The method can furthermore be used as a tool to encourage patients towards a more active lifestyle and to evaluate interventions.

## Competing interests

The authors declare that they have no competing interests.

## Authors' contributions

CW conceived the study, coordinated the execution of the study, evaluated data and drafted the manuscript. TLS, AH and DR conceived and designed the study. MB, CM, MR and TS were involved in the execution of the study. All authors contributed to writing the manuscript and read and approved the final manuscript.

## Pre-publication history

The pre-publication history for this paper can be accessed here:

http://www.biomedcentral.com/1471-2474/11/233/prepub
